# Independent Relationship between Amyloid Precursor Protein (APP) Dimerization and γ-Secretase Processivity

**DOI:** 10.1371/journal.pone.0111553

**Published:** 2014-10-28

**Authors:** Joo In Jung, Sasha Premraj, Pedro E. Cruz, Thomas B. Ladd, Yewon Kwak, Edward H. Koo, Kevin M. Felsenstein, Todd E. Golde, Yong Ran

**Affiliations:** 1 Center for Translational Research in Neurodegenerative Disease, University of Florida, Gainesville, Florida, United States of America; 2 Department of Neuroscience, University of Florida, Gainesville, Florida, United States of America; 3 McKnight Brain Institute, College of Medicine, University of Florida, Gainesville, Florida, United States of America; 4 College of Pharmacy, University of Florida, Gainesville, Florida, United States of America; 5 Department of Neuroscience, University of California San Diego, La Jolla, California, United States of America; University of S. Florida College of Medicine, United States of America

## Abstract

Altered production of β-amyloid (Aβ) from the amyloid precursor protein (APP) is closely associated with Alzheimer’s disease (AD). APP has a number of homo- and hetero-dimerizing domains, and studies have suggested that dimerization of β-secretase derived APP carboxyl terminal fragment (CTFβ, C99) impairs processive cleavage by γ-secretase increasing production of long Aβs (e.g., Aβ1-42, 43). Other studies report that APP CTFβ dimers are not γ-secretase substrates. We revisited this issue due to observations made with an artificial APP mutant referred to as 3xK-APP, which contains three lysine residues at the border of the APP ectodomain and transmembrane domain (TMD). This mutant, which dramatically increases production of long Aβ, was found to form SDS-stable APP dimers, once again suggesting a mechanistic link between dimerization and increased production of long Aβ. To further evaluate how multimerization of substrate affects both initial γ-secretase cleavage and subsequent processivity, we generated recombinant wild type- (WT) and 3xK-C100 substrates, isolated monomeric, dimeric and trimeric forms of these proteins, and evaluated both ε-cleavage site utilization and Aβ production. These show that multimerization significantly impedes γ-secretase cleavage, irrespective of substrate sequence. Further, the monomeric form of the 3xK-C100 mutant increased long Aβ production without altering the initial ε-cleavage utilization. These data confirm and extend previous studies showing that dimeric substrates are not efficient γ-secretase substrates, and demonstrate that primary sequence determinants within APP substrate alter γ-secretase processivity.

## Introduction

The amyloid β (Aβ) peptide is the core component of senile plaques in Alzheimer’s disease (AD) brains [1], [2], [3]. This peptide is produced from the amyloid precursor protein (APP) by sequential cleavages of β-secretase and γ-secretase [4]. β-Secretase cleavage releases the ectodomain of APP and produces the 99 amino acid membrane-anchored CTFβ. CTFβ is subsequently cleaved by γ-secretase to produce various Aβ isoforms and APP intracellular domain (AICD) fragments [5]. Aβ has multiple isoforms [6], [7]. Aβ40 is typically the major species produced, whereas Aβ37, Aβ38, Aβ39, and Aβ42 are produced at lower levels. Other Aβ isoforms including Aβ34, Aβ41, and Aβ43 are produced under various circumstances [6], [8], [9], [10]. Relative increases in long Aβs (i.e., Aβ42 or Aβ43) are tightly linked to increased risk for AD and biologically related to the increased propensity for these long Aβs to aggregate [11]. Many presenilin *(PSEN)* and *APP* mutations linked to early onset familial AD (FAD) increase the relative amount of Aβ42/Aβ40 in *in vitro* and *in vivo* paradigms [12], [13], [14], [15]. Aβx-42 has been shown to be the earliest form of Aβ in AD brains [16],[17],[18]. Aβ42 has a much stronger tendency to aggregate than Aβ40 [19], [20]. In addition, Aβ42 seeding is essential for parenchymal and vascular amyloid deposition in mice [21]. Aβ43 has similar aggregation properties both *in vitro* and *in vivo* [22], [23].

Elegant studies from Ihara and colleagues that have now been confirmed and extended demonstrate that γ-secretase cleavage of APP occurs initially at ε-sites near the cytoplasmic face of the TMD in CTFβ. Subsequently, variable numbers of step-wise carboxyl peptidase-like cleavages of 3–5+ amino acids combined with different initial ε-cleavage site utilization produce the various levels of the different Aβ peptides [10], [24], [25]. Increased long Aβ can thus arise from altered ε-cleavage site utilization, decreased step-wise cleavage (processivity), or a combination of these two. Multhaup and colleagues have proposed that when CTFβ forms dimers, γ-secretase processivity is reduced due to steric hindrance by interhelical interactions of the tandem TMD GXXXG motifs, leading to increased long Aβ isoforms [26], [27]. Specifically, they reported that artificial mutations that induce CTFβ dimerization increase long Aβ or, alternatively, alterations in the GXXXG motif that inhibit dimerization decreased long Aβ along with increase in short Aβs. Further, they linked this effect on dimerization to mechanisms of action of NSAID based acidic γ-secretase modulators (GSMs) that decrease long Aβ production by increasing γ-secretase processivity [28]. The binding site for GSM can be shown to overlap with the GXXXG motif in APP [28], [29]; they decrease production of long Aβ by promoting processivity and have been shown to reduce dimerization of APP. In some cases, small molecules that inhibit APP homodimerization reduce Aβ40 production along with long Aβ42 although their effects appear to additionally involve inhibition of β-secretase activity [30]. Despite these interesting data that associate dimerization of substrate with increased long Aβ production, multiple studies have not seen this association reporting that dimerization decreases or even blocks substrate cleavage by γ-secretase. An APP protein with a FK506-binding protein (FKBP) fused to the C-terminus of APP could be cleaved normally by γ-secretase [31], however, following addition of the synthetic membrane-permeable drug AP20187, which induced dimerization of the APP-FKBP chimera, showed markedly decreased total Aβ levels due to inhibited γ-cleavage [31]. Dimerization of other γ-secretase substrates such as Notch or Glycophorin A has also been shown to reduce γ-secretase cleavage studied in *Drosophila* [32], [33], [34].

**Figure 1:**
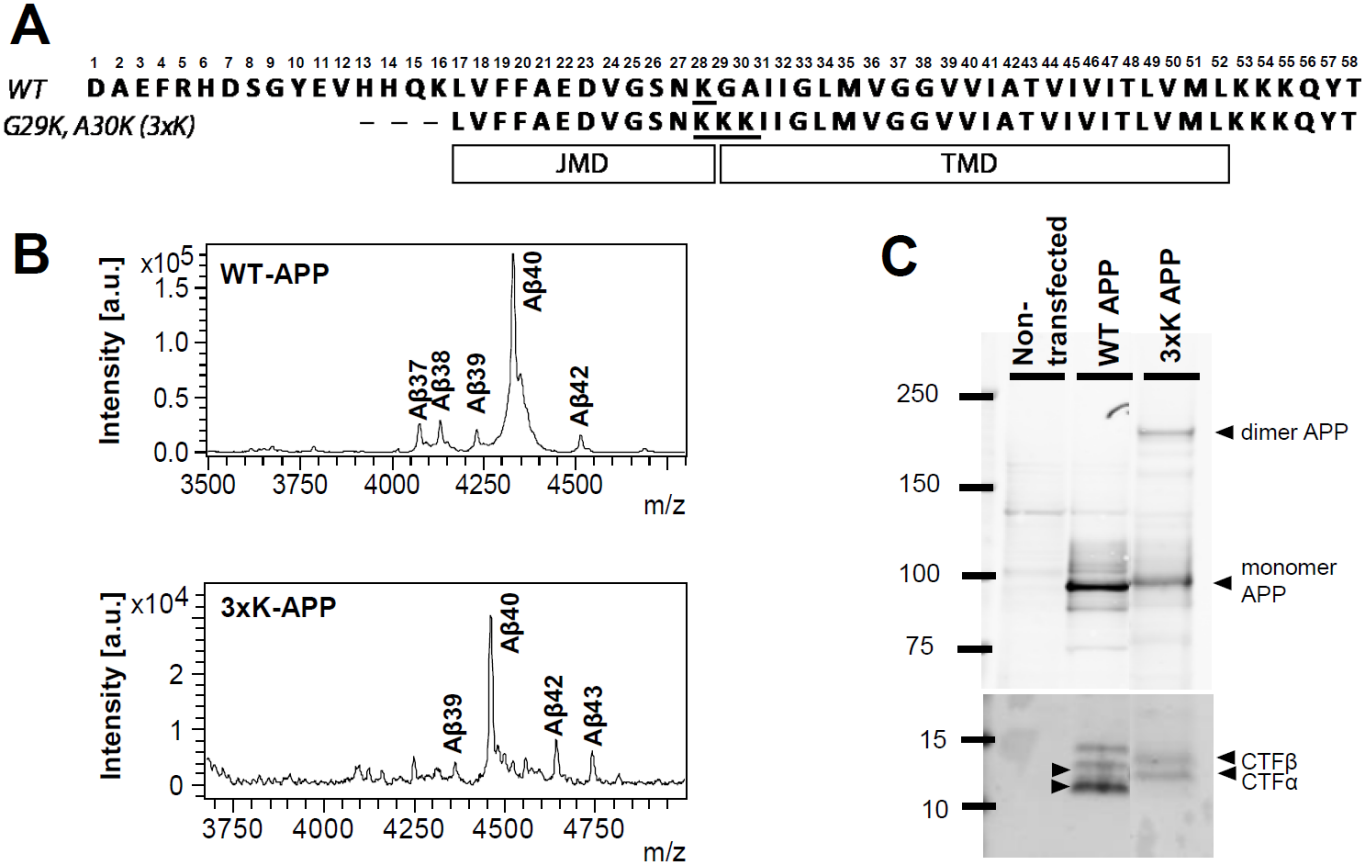
An APP dimer in 3xK-APP mutant-overexpressing CHO cells. (A) Schematic of WT-APP and 3xK-APP mutant. (B) Aβ profile analyzed by IP/MS demonstrated increased Aβ42 and Aβ43 levels of the mutant compared to WT. (C) APP dimer bands migrated at ∼200 kDa along with APP monomer bands at ∼100 kDa in a 3–8% tris-acetate gel. Both WT APP and 3xK-APP were normally processed to CTFα and CTFβ, but 3xK-APP produced less CTFα and CTFβ than WT-APP (Also see Figure S1 in File S1).

Although we felt the weight of the evidence suggested that APP dimerization reduces or prevents γ-secretase cleavage, an unexpected finding was provocative enough that we decided to revisit this issue. We have recently reported that an artificial APP mutant (referred to as 3xK-APP or G29K/A30K) with three lysines immediately downstream of the juxtamembrane domain (JMD) (See Figure 1A) dramatically increases both Aβ42 and Aβ43 by decreasing γ-secretase processivity [9]. In subsequent studies reported here, we have found that the 3xK-APP formed SDS-stable dimers. Thus, it seemed plausible that the 3xK-APP increased substrate dimerization and this increased the long Aβ isoforms. To directly evaluate the relationship between dimerization and Aβ production, we produced the APP-based substrates, recombinant WT-C100Flag and 3xK-C100Flag substrates, and took advantage of the fact that these substrates form SDS-stable multimers that can be resolved on and purified from SDS-PAGE gels [35]. Monomers, dimers, and trimers of the two recombinant substrates could be isolated and were shown to remain predominantly in their initially purified form after γ-secretase activity assays, indicating little major alterations in aggregation state following the activity assay. Using both ELISA and immunoprecipitation-mass spectrometry (IP/MS) to assess cleavage of these substrates in *in vitro* γ-secretase cleavage assays, we find i) that the dimers and trimers of WT and 3xK substrates are not cleaved efficiently by γ-secretase cleavage and ii) that increased levels of the long Aβ peptides are produced from monomeric 3xK substrate without alterations in ε-site utilization. These studies indicate that alterations in γ-secretase processivity are not attributable to dimerization of substrate, but, rather, dependent on primary sequence of the substrate.

## Materials and Methods

### Cell culture

Chinese hamster ovary cells (CHO) stably overexpressing APP695wt and G29K/A30K(3xK)-APP695wt [9] were grown in Ham’s F-12 medium (Life Technologies) supplemented with 10% fetal bovine serum and 100 units/ml of penicillin and 100 µg/ml streptomycin. Cells were grown at 37°C in a humidified atmosphere containing 5% CO_2_ in tissue culture plates (Costar). The cells were harvested at confluence, and then utilized for biochemical analyses.

### Western Blotting

Each of the WT-APP and the 3xK-APP expressing cells were harvested and lysed in radioimmunoprecipitation assay (RIPA) buffer (Tris-HCl, pH 7.4 50 mM, NaCl 150 mM, Triton X100 1%, Sodium deoxycholate 0.5%, SDS 0.1%) [36]. For APP dimer/monomer detection, we utilized 3–8% Tris-Acetate gels (Biorad) and for CTF detection we used 10% Bis-Tris gels (Biorad). The lysates were subsequently used for immunoblotting and detection of full-length APP and carboxyl terminal fragments (CTFs). APP C-terminal specific polyclonal antibody A8717 (1∶500) (Sigma-Aldrich) was used for the APP and CTFα/β detection. For recombinant substrate detection, we used 4–12% Bis-Tris gels (Biorad) and detected with monoclonal 6E10 antibody (1∶1000) (Covance, Emeryville, CA, USA). The blots were developed using an Odyssey infrared scanner (LiCor Biosciences, Lincoln, NE, USA).

### Mass Spectrometry of Aβ

Conditioned media from WT-APP and 3xK-APP cells were harvested and used to analyze secreted Aβ profiles using matrix-assisted laser desorption/ionization time of flight (MALDI-TOF) mass spectrometry analysis. Secreted Aβ peptides from conditioned media were analyzed as previously described with the following modifications [37], [38], [39]. Briefly, the Aβ peptides were immunoprecipitated using Ab5 recognizing the Aβ1-16 epitope [40] and sheep anti-mouse IgG magnetic Dynabeads (Life Technologies, catalog no. 11201D) and eluted with 0.1% trifluoroacetic acid (TFA) in water. Eluted samples were mixed 2∶1 with saturated α-cyano-4-hydroxycinnamic acid (CHCA) matrix (Sigma) in mixture of acetonitrile (60%) and methanol (40%) and loaded onto a CHCA pretreated MSP 96 target plate-polished steel (Bruker, Billerica, MA, USA - Part No. 224989). Samples were analyzed using a Bruker Microflex LRF-MALDI-TOF mass spectrometer. The theoretical average molecular weights of Aβ and AICD fragments were calculated using ExPASy Compute pI/Mw tool.

### Aβ ELISAs

Monoclonal antibodies to Aβ were generated by the Mayo Clinic Immunology Core facilities (Jacksonville, FL, USA). Ab13.1.1. was raised against Aβ35-40 and is specific for Aβx-40, and exhibits minimal cross-reactivity with other Aβ peptides. Ab 2.1.3 was raised against Aβ35-42 and is specific for Aβx-42. The Aβ38 antibody (Ab38), supplied by P. Mehta (Institute of Basic Research, Staten Island, NY, USA), specifically recognizes Aβx-38 and shows no cross-reactivity with other Aβ peptides. For cell-based screens, Aβ was captured from conditioned medium with either Ab5, Ab38, Ab13.1.1, or Ab2.1.3 (coated at 10–50 µg/ml in EC buffer: 5 mM NaH_2_PO_4_-H_2_O, 20 mM Na_2_HPO_4_, 400 mM NaCl, 2.5 mM EDTA, 150 µM BSA, 800 uM CHAPS, and 8 mM NaN_3_) on Immulon 4HBX Flat-Bottom Microfilter 96-well plates (Thermo Scientific, Waltham, MA, USA). Aβ peptides were detected with HRP-conjugated Ab5 (epitope in the amino terminus of Aβ1-16). Aβ standards (Bachem, King of Prussia, PA, USA) were prepared by dissolving in hexafluoroisopropanol (HFIP) at 1 mg/ml with sonication, dried under nitrogen, resuspended at 2 mg/ml HFIP, sonicated again and dried under nitrogen. The resulting Aβ was resuspended in 0.01% ammonium hydroxide, portioned into aliquots in EC buffer, and frozen at −80°C. Following these steps, the Aβ is monomeric, as determined by size-exclusion chromatography.

### Recombinant Protein Production and Extraction

Recombinant DNAs encoding WT-C100Flag [35], [41] or 3xK-C100Flag [9] were produced and cloned into pET-21b+ vectors (Life Technologies). The proteins were overexpressed and purified from *Escherichia coli* BL21 using HiTrap Q-column (GE Life Science, Little Chalfont, UK). The recombinant proteins were mixed with sample buffer and run on the precast 4–12% Bis-Tris gels (BioRad) for western blotting to determine the size of monomer, dimer, and trimer forms of the proteins. N-terminal specific antibody 6E10 (Covance, Gaithersburg, MD) was used to detect the oligomers of the recombinant proteins. After the size is determined, the monomer, dimer, and trimer of each protein are directly excised from the preparative gels that were loaded with our protein samples. Each of the excised gel parts was pestled in 150 mM Sodium Citrate buffer (pH 6.8). The supernatant containing the elute is used for further validation of oligomeric state by rerunning them on 4–12% Bis-Tris gels and stained using Coomassie blue solution and immunoblotted with Ab5 antibody. The concentration of monomer, dimer, and trimer was determined using BCA assays (Thermo Scientific). The eluates were further utilized for *in vitro* γ-secretase assay.

### *In vitro* γ-secretase assay

For the *in vitro* γ-secretase assay, 25 µg/mL of each substrate was incubated with the membrane containing γ-secretase [42] (100 µg/ml total proteins) in 150 mM sodium citrate buffer, pH 6.8, containing complete protease inhibitor (Roche, Indianapolis, IN, USA) for 2 hours at 37°C. The Aβ peptides were captured using Ab5 bound to sheep anti-mouse IgG magnetic Dynabeads. The AICD fragments were captured using anti-FLAG M2 magnetic beads (Sigma). Anti-FLAG M2 magnetic beads are anti-FLAG M2 (mouse monoclonal) antibody attached to superparamagentic iron and then bound to 4% agarose beads. The M2 antibody recognizes FLAG sequence (DYKDDDDK) at the N-terminus, (Met-N-terminus) and C-terminus. The beads were then washed with water and the fragments eluted using 0.1% TFA (Thermo Scientific) in water. The eluted fragments were further processed for MS, as described above.

### Statistical analysis

*In vitro* data were expressed and graphed as the mean±SEM using GraphPad Prism 5 software. Analysis was by two-way analysis of variance (ANOVA) followed by bonferroni post-hoc testing for group differences. The level of significance was set at *p*<0.05 in all tests.

## Results

### 3xK-APP Mutant Dimerization in CHO Cells

In our previous studies examining the interaction between substrate sequence and GSM activity, we have found that an artificial APP mutant (referred to as 3xK-APP) with three lysines immediately downstream of the JMD in APP dramatically increases both Aβ42 and Aβ43 by decreasing γ-secretase processivity [9]. Figure 1A shows JMD-TMD sequences of WT-APP and 3xK-APP CTFβ, and representative Aβ profiles from IP/MS study of WT-APP and 3xK-APP are shown in Figure 1B. Subsequently, Western blot analysis of the 3xK APP extracted from a stable CHO cell line revealed the expected APP monomeric holoprotein migrating at molecular weight ∼100-∼120 kDa, as well as a species consistent with a holoprotein dimer migrating at ∼200+ kDa weight (Figure 1C). This 200+ kDa APP species was not observed in a CHO cell line overexpressing WT APP. As previously reported, 3xK-APP was processed into CTFα and CTFβ; though both bands migrate slower on SDS-PAGE than the WT CTFs (Figure 1C). The Western blot in Figure 1C was probed with anti-APP C-terminal antibody (A8717) that detects both CTFα and CTFβ. Furthermore, we explicitly indicate CTFβ expression for WT-APP and 3xK-APP by using two Aβ N-terminal specific antibodies, 6E10 and 82E1 (Figure S1 in File S1). Consistent with the observation in CTFβ detected with A8717 antibody (Figure 1C), 3xK-CTFβ bands probed with 6E10 and 82E1 antibodies showed slower migration on SDS-PAGE than the WT-CTFβ (Figure S1 in File S1).

### Dimers and Trimers of Recombinant APP substrate Decrease Aβ Production

**Figure 2:**
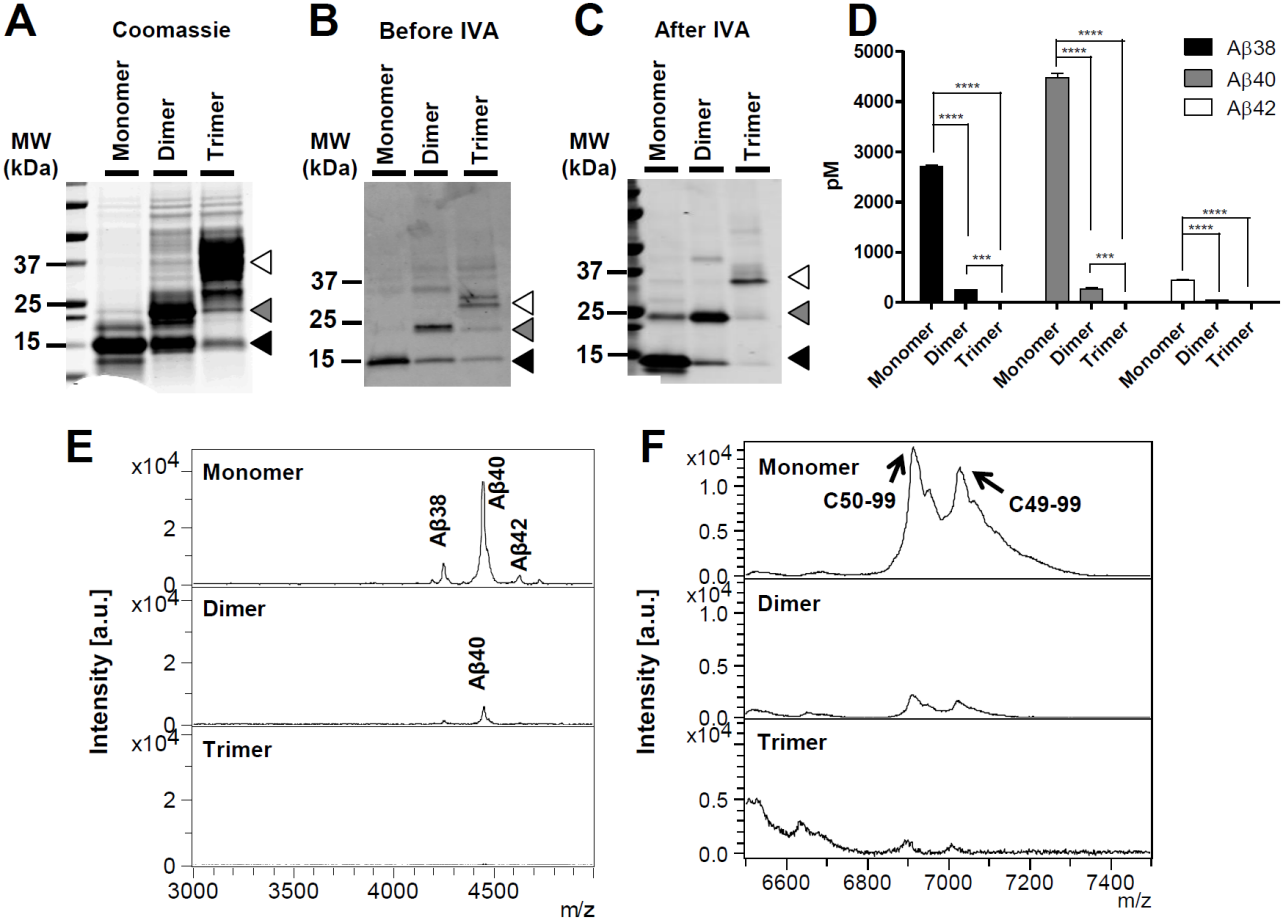
Purified recombinant WT-C100Flag dimer and trimer showed reduced Aβ production. (A) The purified recombinant WT-C100Flag monomer, dimer, and trimer eluates were loaded on a SDS-PAGE gel. WT-C100Flag formed into SDS-stable monomer, dimer, and trimer shown by Coomassie staining. Monomer band was identified at ∼12 kDa (closed arrow), dimer band was identified at ∼24 kDa (grey arrow), and trimer band was at ∼36 kDa (open arrow). (B) Monomer, dimer, and trimer WT-C100Flag extracted from a preparative gel were evaluated by Western blotting with 6E10 antibody before and after IVA. For IVA, the equal concentration of monomer, dimer, and trimer substrate was utilized (25 µg/mL). (C) The purified substrates maintained largely remain as monomer, dimer or trimer following IVA, with an exception that a small amount of monomer aggregated into dimer. (D) The concentrations of Aβ peptides generated during an *in vitro* γ-secretase assay were measured using sandwich ELISAs. Aβ38, Aβ40 and Aβ42 levels decreased in dimer compared to monomer, and the Aβ levels for trimer were under detection limits. (D) Aβ and (E) AICD profiles of C100Flag monomer, dimer, and trimer are characterized using IP/MS analyses. For identification of Aβ species and AICD fragments, the calculated mass is compared with the observed mass, italicized in parentheses: For monomeric substrate, Aβ38 is 4261.064 (*4262.78*), Aβ40 is 4457.945 (*4461.05*), Aβ42 is 4640.108 (*4645.29*), and AICD50-99 is 6916.021 (*6905.66*), AICD49-99 is 7028.590 (*7018.82*). For the dimeric substrate, Aβ40 is 4459.094 (*4461.05*) and AICD50-99 is 6913.934 (*6905.66*) and AICD49-99 is 7026.387 (*7018.82*). This experiment was repeated 3 times with 2–3 replicates each time. Results were analyzed by two way analysis of variance (ANOVA) followed by bonferroni post hoc testing (****p<0.0001, ***p<0.001).

Given conflicting data in the literature as to whether a dimeric substrate could be cleaved by γ-secretase and account for shifts in processivity of γ-secretase towards longer Aβ peptides, we directly tested if decreased γ-secretase processivity resulting from long Aβ production is influenced by substrate dimer or trimer formation using an *in vitro* system. Recombinant WT-C100Flag that serves as an APP substrate akin to CTFβ was used for γ-secretase cleavage assay after additional extraction steps for obtaining its monomer, dimer, and trimer forms. One notable characteristic of this recombinant substrate is the ability to aggregate rapidly and form SDS stable multimers that can be resolved by SDS-PAGE (Figure S2A in File S1) [35]. Figure S2A in File S1 illustrates WT-C100Flag monomer and multimer substrates analyzed by Western blot using a CTFβ detecting antibody (6E10) after SDS-PAGE. Following a preparative SDS-PAGE, we purified WT-C100Flag monomers, dimers, and trimers by directly cutting and eluting the bands from the gel at ∼12 kDa (monomers), ∼24 kDa (dimers), and ∼36 kDa (trimers) (Figure 2A). The concentrations of the eluates measured from monomer, dimer, and trimer substrates were 1.6 mg/mL, 1.8 mg/mL, and 0.9 mg/mL, respectively. Following elution from the gel, purity and stability were then assessed by running the purified proteins on SDS-PAGE and Western blotting following purification and after incubation in an *in vitro* γ-secretase cleavage assay (IVA) (Figure 2B, 2C). The eluates were used at 25 µg/mL for IVA. When initially purified the monomer remains as monomer, the dimer partially dissociates into monomer and aggregates further; the purified trimer also aggregates further and dissociates into dimer and monomer. This pattern was also observed following the IVA; with the exception that monomer forms a small amount of dimer. Following the IVA, from each group, Aβ levels were measured by specific Aβ ELISAs (Figure 2D), and Aβ and AICD profiles were analyzed by IP/MS (Figure 2E and 2F). As assessed by ELISA, Aβ production was significantly decreased when WT-C100Flag dimers and trimers were used as substrates. Notably, purified monomer was a much more efficient substrate than non-purified substrate. In multiple assays, the purified substrate was cleaved ∼10–∼14 times more efficiently. Further, IP/MS and ELISA showed no evidence for alterations in Aβ profiles; all species diminished equally (Figure 2D and 2E). Similarly, when assessed by IP/MS there was no major shift in ε-site utilization (Figure 2F). Although our results could be confounded by challenges in detecting aggregates of the cleavage products, we do not think that this is the case. The ELISAs employed efficiently recognize various Aβ aggregate assemblies [18]. Thus, it is unlikely that dimeric or trimeric substrates are generating dimeric or timeric cleavage products that are escaping detection. As to whether the Aβ generated from the dimeric substrate remains dimeric is challenging to address as the solvents used for IP/MS studies could dissociate a dimer into monomers. This would also be challenging to address by gel-based methods given the large amounts of substrate used and the potential for artifactual dimerization of Aβ [43]. As the purified dimer appears capable of dissociating into monomer, the simplest explanation for this observation is that γ-secretase cleaves monomer substrate following dissociation of the dimer.

### Effect of 3K-C100Flag on Aβ42 production is independent of its dimerization

**Figure 3:**
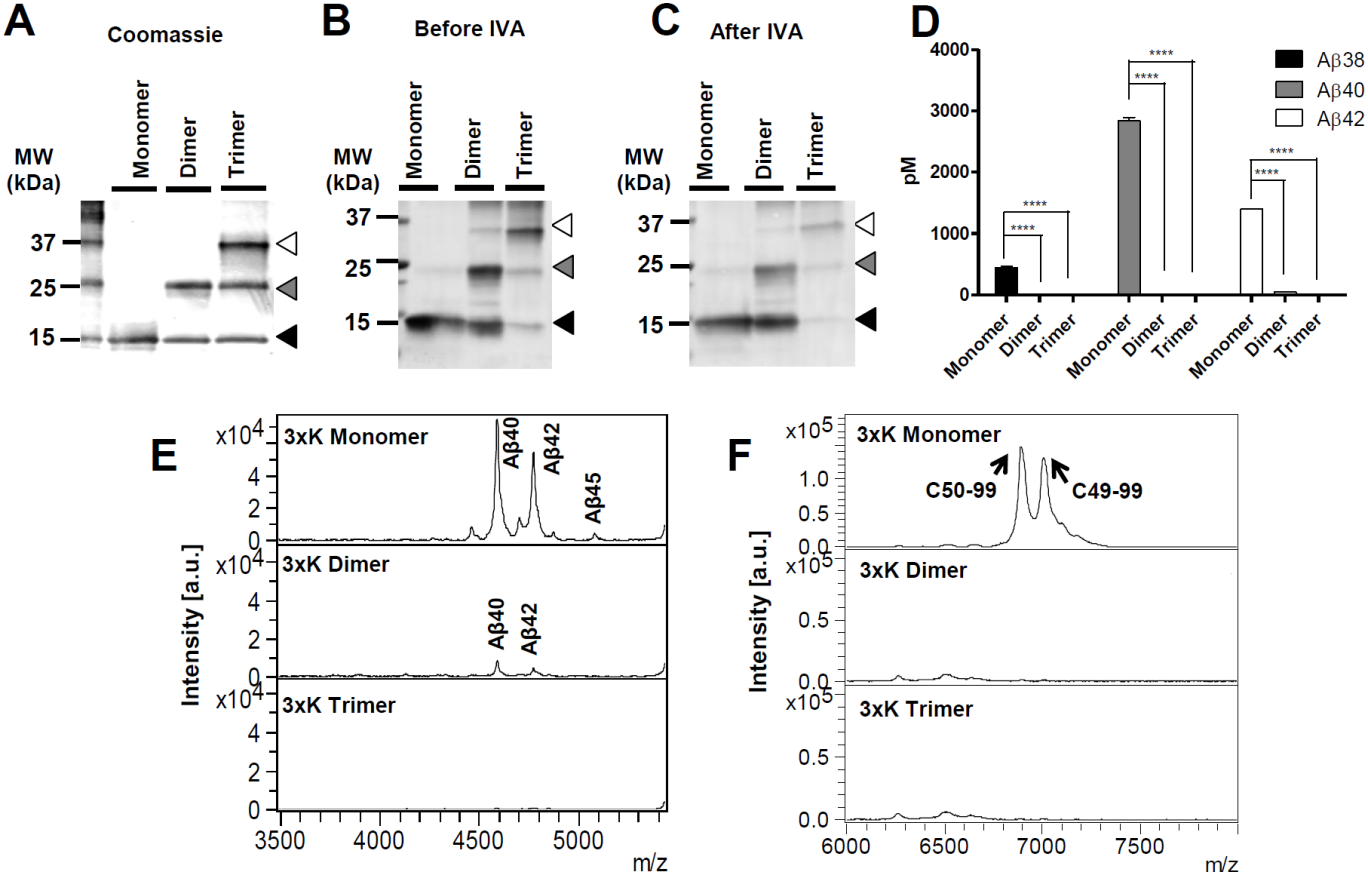
Recombinant 3xK-C100Flag monomer increased longer Aβ peptides. (A) The purified monomer, dimer, and trimer of recombinant 3xK-C100Flag were loaded on a SDS-PAGE gel and identified from the gel stained by Coomassie blue. The size of the monomer was at ∼12 kDa (black arrow), the dimer was at ∼24 kDa (grey arrow), and the trimer was at ∼36 kDa (open arrow). (B–C) Western blot of the purified monomer, dimer, and trimer 3xK-C100Flag substrates before/after IVA did not show significant changes in relative amounts of the various multimers. (D) Aβ ELISAs showed that Aβ production measured from 3xK-C100Flag dimer substrate is significantly reduced compared to that from 3xK-C100Flag monomer, but that there is no evidence for major shifts in Aβ ratios (E). Aβ profiles generated from IP/MS of the media show that for 3xK-C100Flag there is increased relative production of longer Aβ isoforms compared to WT substrate (see Figure 2). Molecular mass (m/z) of monomeric 3xK-C100Flag for Aβ40 is 4588.43 (calculated m/z: 4589.27), for Aβ42 is 4773.07 (calculated m/z: 4773.51), and for Aβ45 is 5084.52 (calculated m/z: 5086.90). 3xK-C100Flag dimer and trimer showed decreased Aβ levels compared to the monomer. Molecular mass of dimeric 3xK-C100Flag for Aβ40 is 4587.48 (calculated m/z: 4589.27) and for Aβ42 is 4769.235 (calculated m/z: 4773.51). (F) AICD profiles show that C49-99 and C50-99 are the dominant peaks for 3xK-C100Flag monomer and that this profile is similar to that observed for WT substrate. Molecular mass (m/z) of monomeric 3xK-C100Flag for AICD50-99 is 6915.460 (calculated m/z: 6905.66), for AICD49-99 is 7027.141 (calculated m/z: 7018.82). AICD production from 3xK-C100Flag dimer and trimer is markedly decreased in comparison to monomer. This experiment was performed twice with duplicates. Results were analyzed by two-way analysis of variance (ANOVA) followed by bonferroni post hoc testing (****p<0.0001).

Given these findings, we tested whether 3xK-APP substrate can increase long Aβ peptides without dimerization. As with non-purified WT-C100Flag substrate (Figure S2A in File S1), non-purified 3xK-C100Flag substrate rapidly aggregated and formed SDS-stable multimers on SDS-PAGE gels. 3xK-C100Flag substrate monomers and multimers were further detected by Western blot (Figure S2B in File S1). Using methods described above, monomer, dimer and trimmer of 3xK-C100Flag were purified and analyzed after purification and IVA (Figure 3A–3C). The concentrations of the 3xK-C100Flag eluates measured from monomer, dimer, and trimer substrates were 0.95 mg/mL, 0.9 mg/mL, and 1.2 mg/mL, respectively. As for 3xK-C100Flag, 25 µg/mL of each eluate was utilized for the IVAs. Analysis of Aβ production and ε-cleavage site showed that as observed with the WT substrate, multimerization of the mutant substrate impedes γ-secretase cleavage. Aβ levels measured from 3xK-C100Flag dimer and trimer were significantly less than its monomer when analyzed by Aβ specific ELISAs (Figure 3D). This was further confirmed by Aβ and AICD profiles illustrated from IP/MS. 3xK-C100Flag monomer increased the relative production of long Aβ species, Aβ42 and Aβ45, without significantly altering ε-site utilization (Figure 3E, 3F). Notably, the mutant monomer showed no evidence for aggregation after the IVA (Figure 3C). As discussed for the WT-C100Flag substrate, the Aβ produced from the 3xK-C100Flag dimer substrate could arise from γ-secretase cleavage of 3xK-C100Flag dimers or dissociating 3xK-C100Flag monomers.

## Discussion

Previous studies have yielded conflicting results regarding the effects of substrate dimerization on γ-secretase cleavage of APP. Here we have revisited this issue with purified recombinant monomeric, dimeric, and trimeric substrates. Under conditions in which monomeric substrate is cleaved efficiently by γ-secretase *in vitro*, the dimeric and trimeric substrates are cleaved very inefficiently. Following purification of the dimeric and trimeric substrates, we present evidence that these assemblies can, to some degree, dissociate into monomer based on their non-covalent interactions; thus, we attribute the minor amount of γ-secretase cleavage of the dimer and trimer not to cleavage of dimer or trimer but to cleavage of monomer following disassociation; however, we cannot completely exclude the possibility that multimers are cleaved by γ-secretase with decreased efficiency. Further, we find no evidence that APP substrate dimers, even if they were cleaved by γ-secretase, alter γ-secretase processivity. The profiles of Aβ peptides generated during of the IVA of a dimeric substrate are not different from monomer. We show that effects of the 3xK-APP mutant substrate that does decrease γ-secretase processivity are observed when the substrate is monomeric. Thus, although this mutant promotes apparent dimerization of APP and increases long Aβ production in cells, the effects of the mutation on APP dimerization and increase in long Aβ appear mechanistically unrelated.

We chose to use an *in vitro* system where we could better control and monitor the aggregation state of the input substrate. We used a gel-purification method that generated well-characterized recombinant monomer and multimer substrates for γ-secretase activity assays. Although we did not rigorously attempt to address structure of our substrates, there is evidence that α-helical conformation of the substrate critical for its dimerization is well preserved in the presence of SDS [35], [44]. Further we find that gel-purified C100Flag monomer is much more efficiently cleaved than non-gel purified substrate. Our studies are consistent with studies using mammalian cell based or *Drosophila* systems that have previously shown that γ-secretase does not cleave substrate dimers or at least does not cleave them efficiently [32], [34]. Thus, these previous findings and our current findings are collectively inconsistent with the hypothesis that dimerization simply decreases γ-secretase processivity. Even the most direct data supporting the relationship between APP substrate dimerization and increased Aβ42 production (i.e., inducing disulfide linkage with K28C mutation) [27] could not be independently confirmed [31]. Hence, the changes in Aβ peptides reported by Multhaup and colleagues may be attributable to the effects of the cysteine mutation on processivity of the monomeric substrate [27], rather than a consequence of dimerization. The effect of dimerization was also linked to the mechanism of action for NSAID-based GSMs and iGSMs. It is an appealing hypothesis that some GSMs that can bind at or near the tandem GXXXG motifs [28], [29] in APP can modulate cleavage by inhibiting dimer formation [28]; however, given our studies showing that amino acid sequence of monomeric substrate determines γ-secretase processivity, GSMs likely modulate the property of the monomeric substrate to enhance γ-secretase processivity. Further, if the homodimerization competes with GSM binding, it is difficult to explain the allosteric models of GSM activity through GSM’s γ-secretase complex binding [45], [46], [47], [48], [49] or possible tripartite interactions between γ-secretase, CTFβ-APP, and GSMs [9].

Although these and other data show that APP substrate dimerization is dissociable from effects on γ-secretase processivity, there remains an intriguing parallel between factors that alter dimerization and factors that alter γ-secretase processivity. Given the tandem GXXXG motif functions as a glycine zipper to induce dimer formation not only in APP but in other proteins as well, we speculate that mutations like the 3xK-APP may i) anchor the TMD more rigidly within the membrane promoting alignment of the glycine zipper and favoring dimerization or ii) by removing the first of three glycine residues in the zipper promote alignment [50], [51]. It is an intriguing possibility that 3xK-APP holoprotein dimerization could be attributable to disruption of cholesterol binding within the tandem GXXXG motifs [52]; however, our data shows this effect on the holoprotein is likely not related to effects on γ-secretase processivity. Indeed, we did not observe increased multimerization of the recombinant 3xK-C100 FLAG substrate compared to WT substrate; both are highly aggregation prone supporting the assertion that the dimerization observed in the 3xK-APP isolated from cells is due to alterations in interactions of the TMD and lipids bilayer.

It is now clear that there are multiple different classes of GSMs and that different classes of GSMs may have different primary binding sites within the γ-secretase complex [53]. The current studies are most directly relevant to NSAID-based GSMs that can bind the tandem GXXXG motif in APP and alter processivity and dimerization [28], [29] in what appears to be an independent fashion. In contrast, a mechanism of GSM action through primary binding to APP [28], [29] would be difficult to reconcile with second generation GSMs that have been shown to bind PSEN or PEN-2 [45], [47], [49], [54], [55]. More generally, given the ample evidence for influence for substrate dependent effects of both acidic and non-acidic GSMs, we believe that it is over simplistic to view GSM mechanism of action solely based on determination of primary binding site [8], [9], [28], [29], [56]. It is clear that GSMs influence γ-secretase processivity in a complex way that involves both interactions of substrate and enzyme [9]. In conclusion, we found that decreased γ-secretase processivity is related to intrinsic properties of the substrate rather than to the APP dimer induced by 3xK-APP mutation. Although reports from one group claim that dimeric substrates lead to decrease in γ-secretase processivity [26], [28], the weight of published evidence suggests that this is not the case [31], [32], [34], [57]. Our data offer both a positive example (mutant monomer is efficiently cleaved and shifts processivity) and reinforce the negative example (dimer/trimer are not cleaved or are cleaved very inefficiently). Collectively, these data suggest that APP dimer formation and γ-secretase processivity are dissociable; thus, they have important implications for design and screening of GSMs as therapeutic agents for AD.

## Supporting Information

File S1 **Figures S1 and S2. Figure S1. The CTFβ of WT- and 3xK-APP stably expressed into CHO cells.** The Western blot analysis shows the CTFβ expression in CHO cells probed with 6E10 and 82E1 antibodies. CTFβ bands from the 3xK-APP show lower expression and migrated slower than the WT-APP. α-Tubulin and β-actin are provided as loading controls. **Figure S2. Purified recombinant WT-C100Flag and 3xK-C100Flag form SDS-stable multimers analyzed by Western blot.** WT-C100Flag substrate and 3xK-C100Flag substrate were loaded on SDS-PAGE gels. The formation of (A) WT-C100Flag and (B) 3xK-C100Flag multimers was shown by Western blot analysis using 6E10 antibody that probes CTFβ following SDS-PAGE. Monomer (black), dimer (grey), and trimer (white) were indicated with arrows.
(DOCX)
