# Supplementary figures and images for: Independent Relationship between Amyloid Precursor Protein (APP) Dimerization and γ-Secretase Processivity
Source: PLoS One. 2014 Oct 28;9(10):e111553. doi: 10.1371/journal.pone.0111553 (PMC4211736; doi:10.1371/journal.pone.0111553)

Supporting Information S1


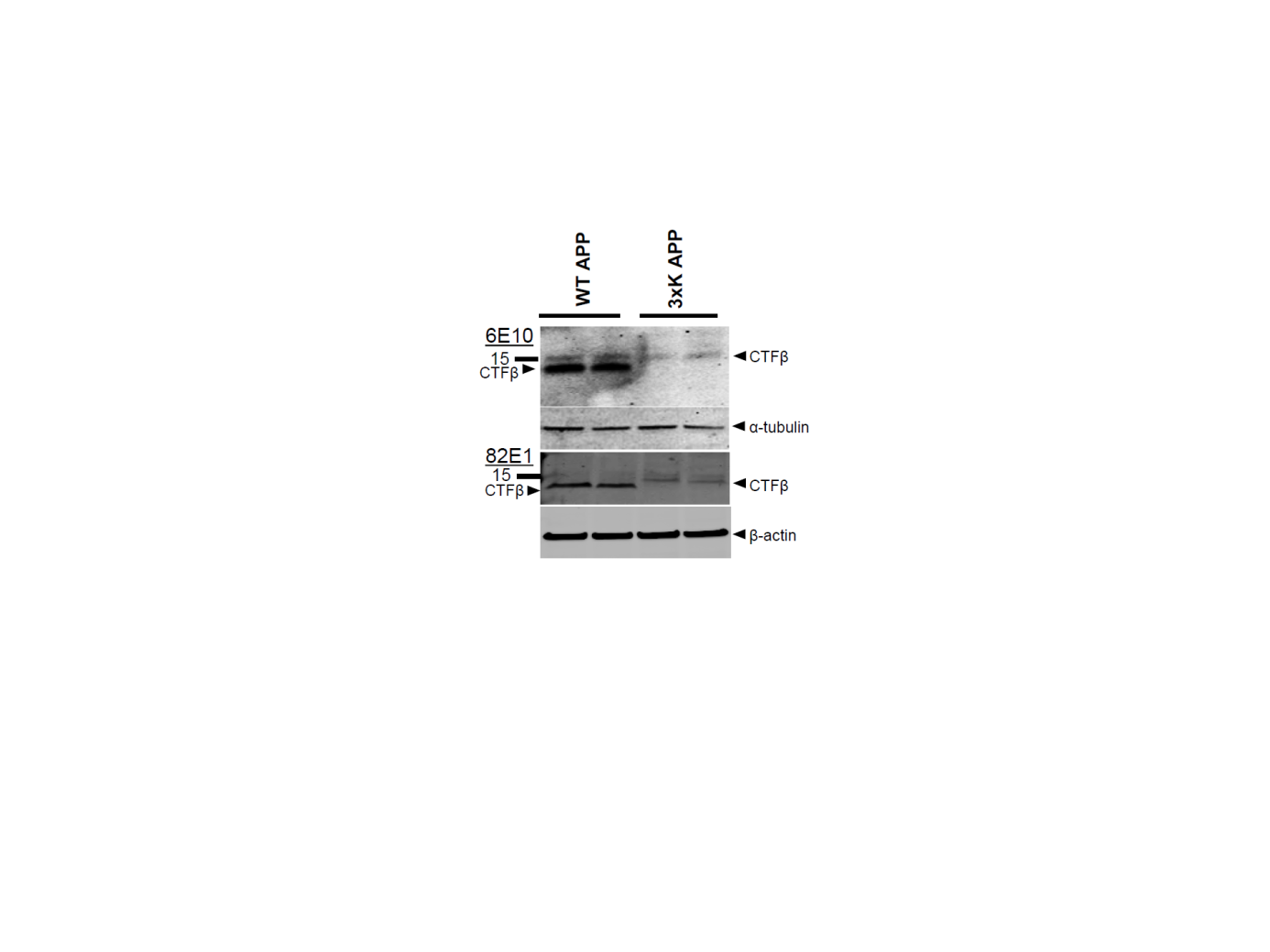


**Figure S1.**


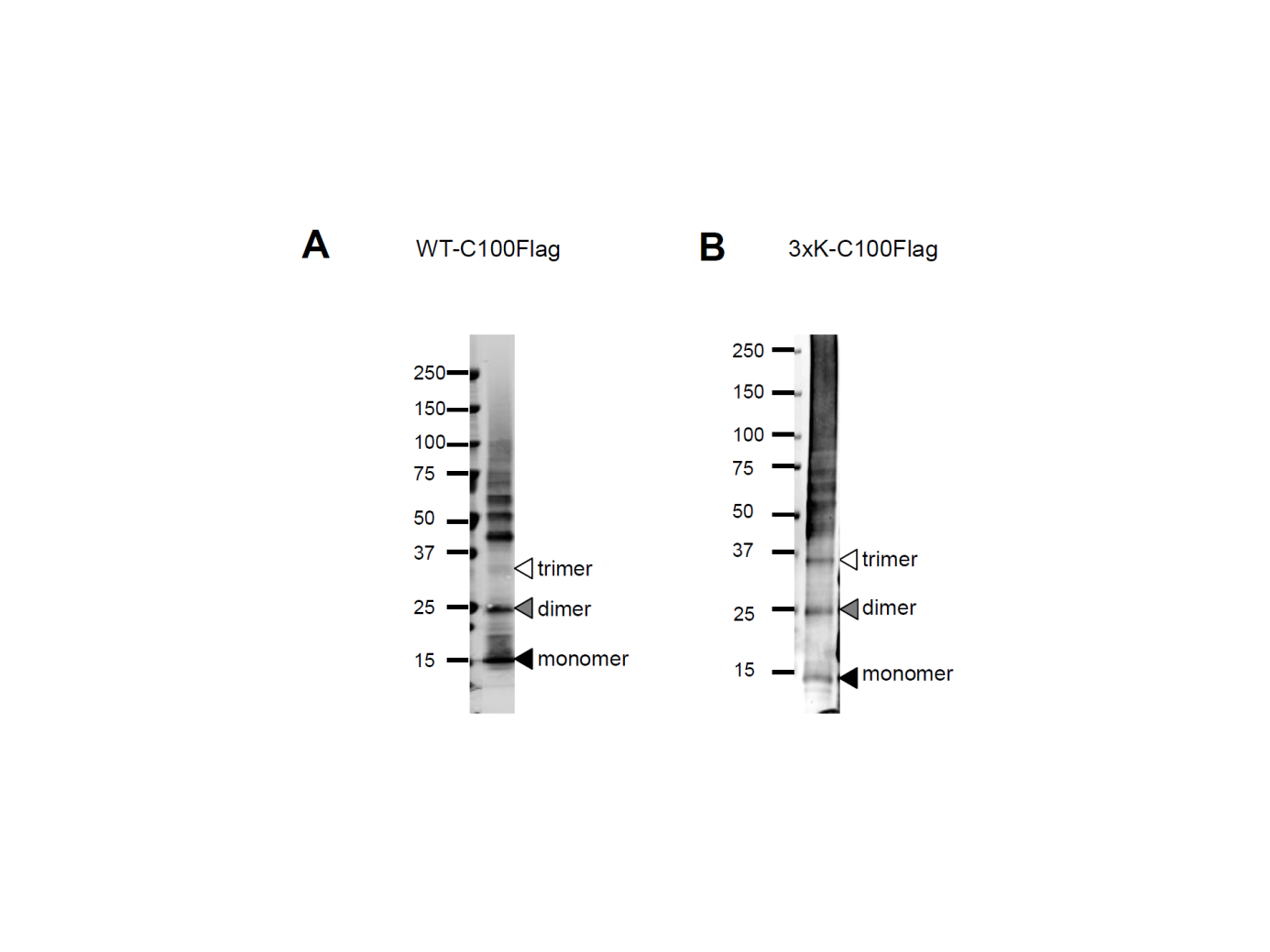


**Figure S2.**

Supplement: File S1 — Figures S1 and S2. Figure S1. The CTFβ of WT- and 3xK-APP stably expressed into CHO cells. The Western blot analysis shows the CTFβ expression in CHO cells probed with 6E10 and 82E1 antibodies. CTFβ bands from the 3xK-APP show lower expression and migrated slower than the WT-APP. α-Tubulin and β-actin are provided as loading controls. Figure S2. Purified recombinant WT-C100Flag and 3xK-C100Flag form SDS-stable multimers analyzed by Western blot. WT-C100Flag substrate and 3xK-C100Flag substrate were loaded on SDS-PAGE gels. The formation of (A) WT-C100Flag and (B) 3xK-C100Flag multimers was shown by Western blot analysis using 6E10 antibody that probes CTFβ following SDS-PAGE. Monomer (black), dimer (grey), and trimer (white) were indicated with arrows. (DOCX) [file pone.0111553.s001.docx]
